# Serine protease inhibitor disrupts sperm motility leading to reduced fertility in female mice^†^

**DOI:** 10.1093/biolre/ioaa049

**Published:** 2020-04-18

**Authors:** Brooke E Barton, Jenna K Rock, Anna M Willie, Emily A Harris, Ryan M Finnerty, Gerardo G Herrera, Prashanth Anamthathmakula, Wipawee Winuthayanon

**Affiliations:** School of Molecular Biosciences, Center for Reproductive Biology, College of Veterinary Medicine, Washington State University, Pullman, Washington, USA

**Keywords:** protease inhibitor, contraceptive, spermicide, sperm motility, fertility, semen liquefaction, vaginal toxicity

## Abstract

Inhibition of the sperm transport process in the female reproductive tract could lead to infertility. We previously showed that a pan-serine protease inhibitor, 4-(2-aminoethyl)benzenesulfonyl fluoride (AEBSF), blocked semen liquefaction in vivo and resulted in a drastic decrease in the number of sperm in the oviduct of female mice. In this study, we used a mouse model to test the efficacy of AEBSF as a reversible contraceptive, a sperm motility inhibitor, and a spermicide. Additionally, this study evaluated the toxicity of AEBSF on mouse vaginal tissues in vivo and human endocervical cells in vitro. We found that female mice treated with AEBSF had significantly less pups born per litter as well as fertilization rates in vivo compared to the vehicle control. We then showed that AEBSF reduced sperm motility and fertilization capability in vitro in a dose-dependent manner. Furthermore, AEBSF also exhibited spermicidal effects. Lastly, AEBSF treatment in female mice for 10 min or 3 consecutive days did not alter vaginal cell viability in vivo, similar to that of the vehicle and non-treated controls. However, AEBSF decreased cell viability of human ectocervical (ECT) cell line in vitro, suggesting that cells in the lower reproductive tract in mice and humans responded differently to AEBSF. In summary, our study showed that AEBSF can be used as a prototype compound for the further development of novel non-hormonal contraceptives for women by targeting sperm transport in the female reproductive tract.

## Teaser

Blockade of the sperm transport process using a pan-serine protease inhibitor can be developed as a non-hormonal contraceptive for women.

## Introduction

Access to effective and inexpensive contraceptives is a pertinent women’s health issue. Besides tubal ligation and vasectomy, the most effective contraceptives (i.e., intrauterine devices, oral birth control, or birth control implants) have failure rates as low as 1% with correct use, but require a prescription from physicians [1]. However, these options are not readily available for certain groups of women such as those without health insurance or those who live in underserved locations [2]. Women without health insurance are 30% less likely to use prescription contraceptives, leaving these women to resort to over-the-counter (OTC) contraceptives [3]. Moreover, these OTC options have failure rates as elevated as 18% for condoms and 28% for spermicides, remarkably higher than any of their prescriptive counterparts [1]. Notably, a total of 25% failure rate is observed when women used OTC contraceptives compared to 1% or less in women using prescription contraceptives [1].

Unintended pregnancy in the United States remains consistently high, approximately 45% of all pregnancies [4]. Male condoms are the most frequently chosen method of OTC contraception, with 97% use among sexually active teenagers in the United States [5]. This same trend appears in developing and underdeveloped countries. In a recent report, in the Republic of Congo, 20% of men used contraception at intercourse, of which 18% used male-controlled methods while only 2% used female-controlled methods [6]. A male-dominated contraceptive using pattern is worrisome as condoms have a high failure rate of 18% and requires immediate attention and action [1]. One goal in developing novel contraceptives is to re-align these contraceptive dynamics by improving the efficacy of female-controlled in comparison with male-controlled methods, empowering women in developing countries while creating more effective and accessible alternatives.

Spermicides are of interest as they are one of the OTC methods. The most common active constituent in spermicides is nonoxynol 9 (N9). These spermicides contain 3–12% N9 per application. N9 acts as a detergent that disrupts the membrane integrity of the sperm, attenuating sperm viability [7]. Additionally, spermicides containing N9 are also known to adversely impact the health of cervical, vaginal, and vulvar tissues [8]. Exposure to N9 increases the exfoliation and re-growth of epithelial cell layers and increases inflammation by attracting macrophages and cytokines in mice [9]. By negatively impacting the integrity of epithelial cells, which functions as the first barrier against pathogenic perturbations, women are more likely to contract sexually transmitted infections (STIs). Accordingly, human immunodeficiency virus (HIV) infection frequency was increased in N9 users (16%) compared to the placebo group (12%) [10]. A similar study demonstrated that mice were five times more likely to be infected with human papillomavirus upon exposure to N9 than their non-exposed control littermates [11]. Therefore, the identification of an alternative compound to N9 is an important step toward the goal of improving contraceptive options for women.

Semen liquefaction is the process of altering post-ejaculated semen from a gel-like to a liquefied (watery) state [12]. Semenogelins (SEMG1 and SEMG2) are gel-forming proteins secreted from seminal vesicles that function to immobilize sperm [13]. Prostate-derived serine proteases called kallikreins (KLKs) are responsible for semen liquefaction [14]. KLKs hydrolyze semenogelins resulting in liquefied semen, and subsequently, sperm gain their motility [15]. Liquefied semen allows sperm to travel and reach the ampulla of the oviduct for fertilization [12, 16]. Specifically, the enzyme KLK3 is primarily responsible for the liquefaction process, as mutations in *KLK3* in men are directly correlated with low sperm motility and fertility defects [17].

More than 20 years ago, an in vitro study showed that a small molecule serine protease inhibitor called 4-(2-aminoethyl)benzenesulfonyl fluoride (AEBSF) suppresses the function of KLK3 [18]. Our recent in vivo study showed that upon AEBSF treatment, semen liquefaction defect was observed in female mice mated with fertile male mice [19]. SEMG2 remains uncleaved in the AEBSF-treated group compared to control-treated female mice. Together these liquefaction defects lead to a significant reduction in the number of sperm reaching the oviduct [19]. In the present study, we aim to provide a proof-of-concept to determine whether protease inhibitors could potentially be used as a contraceptive. Herein, we show that AEBSF (1) effectively and reversibly reduced fecundity in female mice, (2) negatively affected sperm motility and viability, and (3) was significantly less damaging to the vaginal epithelium when treated in vivo in mice as well as in human ectocervical cell line in vitro in comparison with N9.

## Results

### Transcervical injection of AEBSF significantly reduces fertility and in vivo fertilization rate in female mice

**Figure 1:**
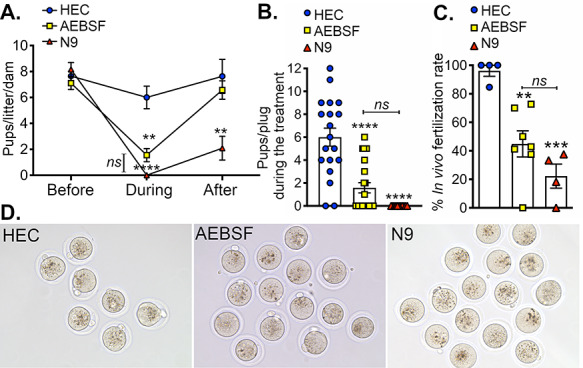
AEBSF reduces the fertility in female mice. (A) Numbers of pups/litter/dam that were born from female mice in vivo “before,” “during,” and “after” transcervical treatments of HEC gel (vehicle control), AEBSF (300 μg in 50 μL HEC gel), and 49 mM (or 3%) N9 (the positive control). ^*^^*^,^*^^*^^*^^*^P < 0.01, 0.0001; compared to their responding group at “before” stage, *ns*; not statistically significant when compared between AEBSF “during” and N9 “during” using a two-way ANOVA with Tukey’s multiple comparisons post-test. *n* = 4–7 mice/treatment/time-point. (B) Number of pups per plug during the treatment. ^*^^*^^*^^*^P < 0.0001; compared to HEC vehicle control, *n =* 4–7 mice/treatment. (C) % fertilization rate in vivo after transcervical treatment of HEC, AEBSF, or N9. ^*^^*^^*^^*^P < 0.0001; compared to HEC vehicle control, *ns*; no statistical differences were seen between AEBSF and N9 fertilization rates using a two-way ANOVA with Tukey’s multiple comparisons post-test. *n =* 4–7 mice/treatment. (D). Images of fertilized eggs in vivo after transcervical treatment of HEC, AEBSF, or N9.

Controlled fertility trials with the use of the contraceptive were performed to determine whether AEBSF effectively attenuates fecundity in female mice in vivo. Prior to the treatment, we ensured that all females exhibited normal fertility by breeding them with a male proven breeder. Females in all treatment groups showed a similar fertility rate of approximately 7.5 ± 0.3 pups/litter/dam (Figure 1A). Hydroxyethylcellulose (HEC) gel was used as a vehicle control. The treatment of HEC gel (50 μL) did not significantly change the number of pups/litters in female mice (6.0 ± 1.3). N9 (3% or approximately 49 mM) was used as a positive control. There were no pups born following N9 treatment. AEBSF (at a dose of 300 μg in 50 μL of HEC gel) significantly lowered the number of pups/litter to 1.6 ± 0.5. There were no statistical differences in pup numbers between the N9 and AEBSF during the treatment. We also showed that the number of pups/plug after AEBSF treatment was consistent with the number of pups/litter/dam (Figure 1B). After discontinuing the treatment of AEBSF, the number of pups born returned to similar levels as before the treatment (6.6 ± 0.7; Figure 1A). After the treatment of N9 was ceased, the number of pups born was severely reduced and significantly less than before the treatment (2.1 ± 1.0). Additionally, we found that the in vivo fertilization rate after AEBSF treatment was at 45% (Figure 1C–D), which is significantly lower than that of HEC-treated controls (96%). Surprisingly, N9 did not completely prevent in vivo fertilization, with a fertilization rate of 20%. These data indicate that AEBSF significantly reduced female fecundity in vivo and its effects were temporary, suggesting its use as an effective and reversible contraceptive.

**Figure 2:**
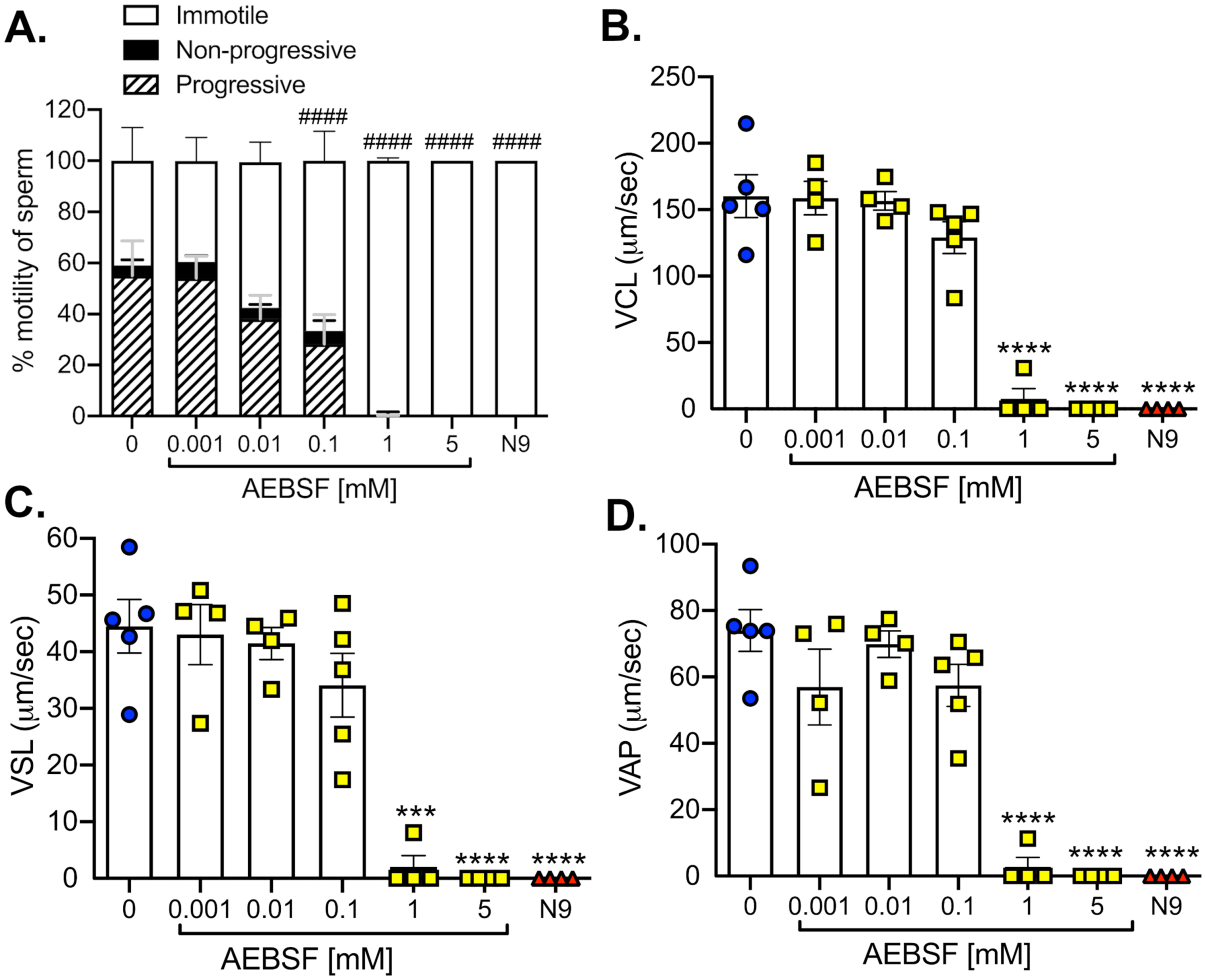
AEBSF disrupts the mouse sperm motility in vitro. Sperm from the cauda epididymis were collected and treated 1 h with various doses of AEBSF and 49 mM N9 (positive control), in comparison with 0 mM AEBSF (vehicle control). Sperm motility was analyzed using SCA. (A) Percentages of immotile, non-progressive motile, and progressive motile sperm are presented. ^####^*P <* 0.0001; % immotile sperm compared to 0 mM, two-way ANOVA, Tukey’s multiple comparisons post-test. (B–D) Sperm velocities after the treatment (μm/s). (B) Sperm curvilinear velocity (VCL), (C) straight line velocity (VSL), and (D) average path velocity (VAP). ^*^^*^^*^,^*^^*^^*^^*^P < 0.001, 0.0001; compared to 0 mM, one-way ANOVA, Sidak’s multiple comparisons post-test. *n =* 4–5 mice/treatment, at least 500 sperm analyzed/treatment/mouse.

### AEBSF decreases the sperm function in mice

We previously showed that AEBSF treatment in female mice led to reduced sperm number in the oviduct [19]. In this study, we aim to evaluate whether AEBSF affects the sperm function by assessing the sperm motility and in vitro fertilization (IVF) rate. To evaluate the sperm motility, sperm extracted from the cauda epididymis were incubated in increasing concentrations of AEBSF for 1 h. In the absence of the treatment (0 mM), approximately 55% of sperm showed progressive motility, 5% were non-progressive and 40% were immotile (Figure 2A, Supplementary Figure 1, and Supplementary Movie 1). AEBSF at doses of 0.1 and 1 mM significantly increased the number of immotile sperm. AEBSF at 5 mM completely inhibited sperm motility with no visible motile sperm, similar to that of N9 at 49 mM (Figure 2A). However, sperm curvilinear velocity (VCL), straight line velocity (VSL), and average path velocity (VAP) were significantly decreased only at 1 and 5 mM of AEBSF treatment (Figure 2B–D). These results suggest that AEBSF attenuates sperm motility in a dose-dependent manner.

**Figure 3:**
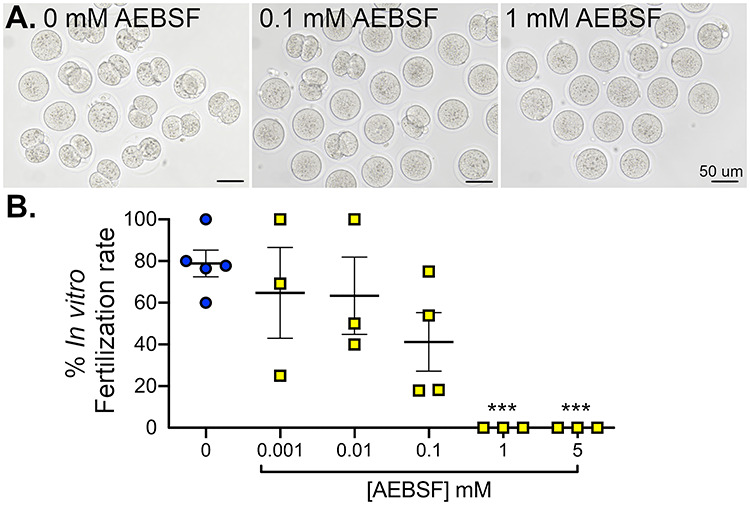
Treatment of AEBSF negatively affected the mouse sperm function in vitro. (A) Images of two-cell embryos 24 h after IVF of eggs and sperm treated with various doses of AEBSF. Scale bars = 50 μm. (B) IVF rate after 8 h indicated by the number of zygotes with male and female pronuclei compared to the total number of eggs in the fertilization dish. Each data point represents eggs from each female mouse. *n =* 31–83 eggs/treatment from a total of 3–5 female mice per treatment condition. ^*^^*^^*^P < 0.001, one-way ANOVA with Sidak’s multiple comparisons post-test.

To determine the effect of AEBSF on sperm function, sperm were incubated in various doses of AEBSF (as described above) before IVF. One hour after incubation, sperm were transferred into a different culture drop containing cumulus–oocyte complexes (COCs) collected from female oviducts. The rate of fertilization was characterized as the number of zygotes to the total number of eggs at 8 h after IVF. Images were taken at 24 h after the IVF, at which zygotes were cleaved to two-cell stage embryos (Figure 3A). Sperm treated with vehicle control showed an average fertilization rate of 78.9 ± 6.4% (Figure 3B). AEBSF caused a significant reduction in fertilization rate in a dose-dependent manner. AEBSF treatment at 1 and 5 mM resulted in a loss of fertilization in vitro (Figure 3B). Therefore, these findings indicate that AEBSF reduced sperm motility and subsequently inhibited the sperm function in vitro.

### AEBSF partly acts as a spermicide

**Figure 4:**
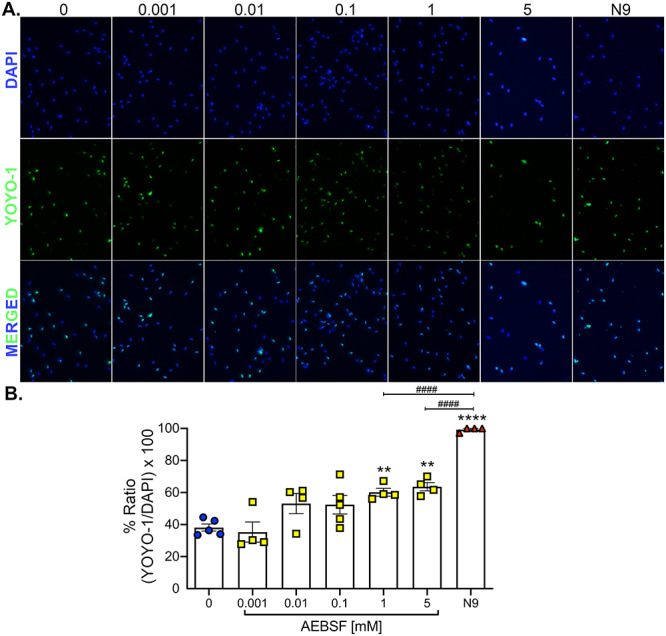
AEBSF exhibits a spermicidal effect in mouse sperm in vitro. (A) Mouse sperm collected from the cauda epididymis were treated for 1 h with various doses of AEBSF or 49 mM N9. Sperm were stained with dead cell dye (YOYO-1) and counterstained with DAPI (nuclei). (B) Signal intensities were quantified and analyzed as a ratio of number of cells with YOYO-1-positive to a total of cells with DAPI-positive per image. Each data point represents sperm from each male. *n =* 4–5 mice/treatment, at least 203–352 sperm analyzed/treatment/mouse. ^*^^*^, ^*^^*^^*^^*^P < 0.01, 0.0001; compared to 0 mM, ^####^*P <* 0.0001; compared to N9, one-way ANOVA, Sidak’s multiple comparisons post-test.

To evaluate whether AEBSF also acts as a spermicide, sperm cell death was evaluated using a YOYO-1 green fluorescent dye. Sperm from the cauda epididymis were treated with AEBSF for 2 h and then immediately incubated with the YOYO-1 dye followed by DAPI staining. N9, a current spermicide, was used as a positive control. AEBSF at doses of 1 and 5 mM increased the percentage of green fluorescent signal compared to a vehicle control (0 mM) (Figure 4). However, only ~ 60% of sperm were dead in 1 and 5 mM AEBSF treatment, unlike N9 which resulted in 99% sperm death. These data suggest that the negative impact of AEBSF on mouse sperm motility and function could partly be due to its spermicidal effect.

### Exposure to AEBSF shows minimal damage to the vaginal epithelium in female mice in vivo but appears to decrease human cervical cell viability in vitro

**Figure 5:**
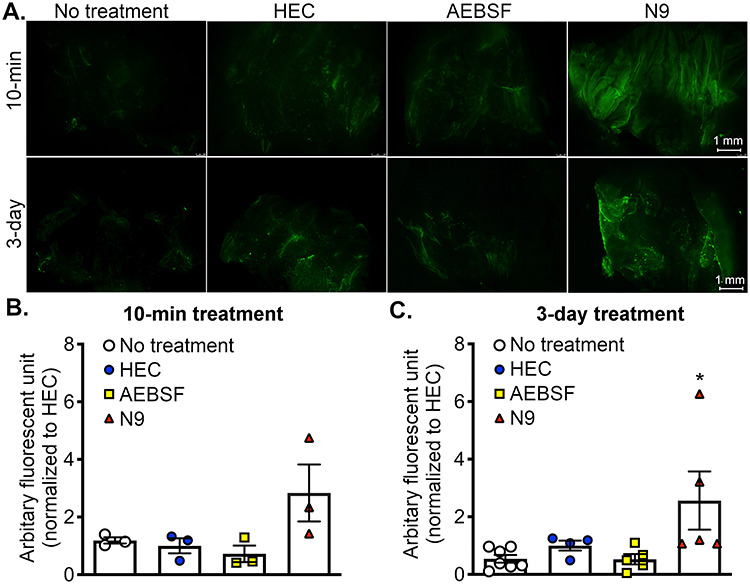
AEBSF did not appear to cause vaginal cell death in vivo in female mice. (A) Green fluorescent images of the mouse vagina after 10-min or 3-day treatment of HEC gel (vehicle control), AEBSF 300 μg in 50 μL HEC gel, or 49 mM N9 (positive control). Vaginal tracts were stained with dead cell dye (YOYO-1). No treatments were used to indicate basal levels of cell death. (B and C) Qualitative analysis of toxicity of AEBSF on vaginal epithelium after (B) 10-min of treatment or (C) daily treatment for 3 consecutive days. Fluorescent signals were normalized to HEC gel. ^*^P < 0.05, one-way ANOVA; Sidak’s multiple comparisons post-test. *n* = 3–7 mice/treatment/time-point.

As N9 is known to be damaging to the vaginal epithelium [8], it is necessary to determine whether AEBSF also caused vaginal toxicity compared to that of N9. Depo-Provera was used to prime female mice to mimic diestrus stage, at which the vaginal epithelial layer is most susceptible to toxicants [20]. In this study, YOYO-1 dye was also used as an indicator for the vaginal cell death. Female mice treated with HEC gel for 10 min or daily for 3 consecutive days showed minimal green fluorescent signal and were comparable to the no-treatment control (Figure 5A). AEBSF at a dose of 300 μg was chosen as it showed a significant effect on female fecundity (Figure 1). Qualitative analysis showed that AEBSF (at either 10-min or 3-day treatment) did not significantly increase the fluorescent signal (Figure 5B–C). As expected, N9 treatment at both time-points significantly increased fluorescent signals compared to other treatment groups.

**Figure 6:**
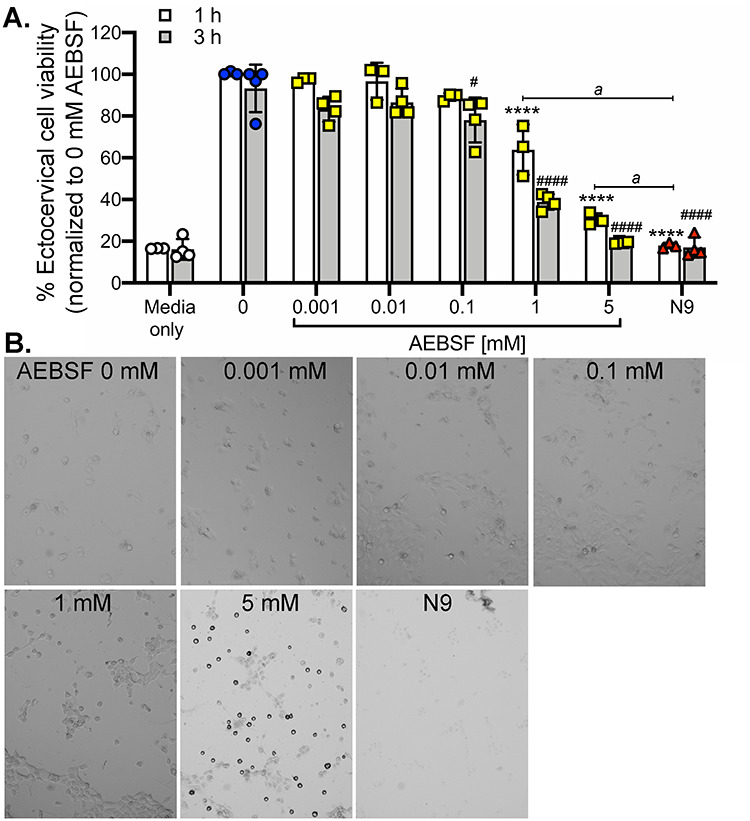
Cell viability of human ectocervical (ECT) cells in vitro after 1 or 3 h after various doses of AEBSF or 49 mM N9 treatment. (A) MTT assay was used to determine the cell viability. The percentage of cell viability was normalized to 0 mM AEBSF (vehicle control). ^*^^*^^*^^*^P < 0.0001; compared to 0 mM at 1 h, ^#^ and ^####^*P* < 0.05 and 0.0001; compared to 0 mM AEBSF at 3 h, *^a^P* < 0.0001; compared to N9 at 1 h. *n* = 3 different experiments repeated in triplicates. Blank data indicate the background readout from wells in the absence of cells. All statistical analysis was determined separately for 1 and 3 h time-points using a one-way ANOVA with Sidak’s multiple comparisons post-test. (B) Bright-field images of cells after 3 h of AEBSF or N9 treatment in comparison with the control (0 mM). All images were taken at 100× magnification.

To determine whether AEBSF exhibits similar trends on human cells as it did in mice, human ectocervical (ECT) cells were used to test the toxicity of AEBSF in vitro. The human ECT cell line was chosen as the ectocervix is the major cell type exposed to vaginal contraceptive gels. AEBSF at doses of 1 and 5 mM significantly decreased ECT cell viability at 1 h and 0.1, 1, and 5 mM of AEBSF reduced cell viability at 3 h (Figure 6A). However, cell viability in groups treated with 1 h of 5 mM AEBSF was significantly different than those treated with N9. Note that 3% N9 significantly decreased cell viability at 1 h to a level as if there were no cells (Blank). Moreover, cellular response to AEBSF was different than that of N9. After 3 h of treatment, cells treated with N9 were lysed and completely devoid of cell structure (Figure 6B). However, cells treated with 5 mM AEBSF appeared to be detached and rounded up from the bottom of the well. This study showed that AEBSF at a dose of 300 μg (equivalent to 25 mM) did not alter the vaginal epithelial cell viability in female mice in vivo, but 1 and 5 mM AEBSF decreased human ECT cell viability in vitro. Regardless, the extent of toxicity of AEBSF was significantly less than that of N9.

## Discussion

Identifying a new OTC contraceptive method that can block semen liquefaction, attenuate sperm motility, reversibly decrease fecundity, and be safer upon exposure to the vaginal epithelium than current comparative OTC options is the main purpose of this study. The prototype compound we identified to address these concerns is a pan-serine protease inhibitor, AEBSF. We acknowledge that the effectiveness of AEBSF on pregnancy prevention is inferior to that of N9 (although not significantly different). This is potentially due to the use of a suboptimal dose of AEBSF in vivo. It is also possible that the higher molarity of N9 gives rise to a superior contraceptive effect as well as a higher vaginal toxicity in vivo and in vitro compared to that of AEBSF. However, our study aims to provide a proof-of-concept that inhibition of sperm transport via blocking liquefaction together with its negative effect on sperm motility and function can be used as OTC female contraceptives. Due to the high dosage of AEBSF used in the mouse model, it is unappealing for pharmaceutical purpose. However, we showed here that using protease inhibitors to block sperm transport can be potentially developed as a novel contraceptive method. Therefore, our following study is to identify new and small molecule inhibitors from available DNA-Encoding Chemistry Technology (DEC-Tec) libraries of 4 billion drug-like molecules with higher efficacy and specificity [21].

Protease inhibitor(s) is also intended to be used in conjunction with a condom. Combining the two contraceptive methods will increase the efficacy of both options (a spermicidal-like gel and a condom) when compared to using alone, thereby potentially lowering failure rates to 1–2.5% [22]. Both male and female condoms are the only birth control method that protect against STIs as well as against pregnancy, a quality that contraceptive gels such as small molecule protease inhibitors do not possess as they are not barrier methods. Therefore, the conjunction of the two methods will protect against STIs and more effectively prevent pregnancy. As an intravaginal suppository gel, protease inhibitors will be a female-controlled method that holds the potential to shape the dynamics of contraceptive use among women, empowering them with an additional method they can utilize for themselves.

AEBSF is known to prevent the cleavage of SEMG proteins through the inhibition of the KLK3 in vitro [18], preventing the semen liquefaction process in vivo [19]. Inhibition of KLK3 is the proposed mechanism through which AEBSF works as a contraceptive. It is valuable to recognize that the KLK3 enzyme is present only after the prostatic fluid is mixed within the ejaculate [14]. In our sperm motility and function analyses, sperm were extracted from the cauda epididymis; therefore, samples are void of prostatic enzymes. Because incubation of mouse sperm with AEBSF effectively inhibited sperm motility, it suggests that AEBSF functions to suppress the sperm function through other mechanisms than inhibiting prostate-derived protease activities. This mechanism may include the inhibition of other proteases present in the epididymal fluid or through a different biochemical process that attenuate sperm motility regardless of the semen liquefaction process.

We showed that AEBSF exhibited spermicidal effects in mice. However, not all sperm treated with AEBSF (at 1 and 5 mM) were nonviable, yet almost all sperm lacked motility and were unable to fertilize the eggs in vitro. These data suggest that the negative impact of AEBSF on mouse sperm motility and function was partially due to its spermicidal effect. Therefore, the inhibitory effect of AEBSF on sperm motility may be through other mechanisms. If AEBSF inhibits the function of other proteases, it is potentially through the disruption of serine protease activity on the sperm. These proteases, located at the acrosomal region, are thought to be involved in the binding of the zona pellucida. However, a knockout mouse model of one of the serine proteases, acrosin (*Acr*^−/−^), shows normal fertility [23]. Nevertheless, male mice with a double deletion of *Acr* and serine protease 21 (*Prss21*), called *Acr*^−/−^;*Prss21*^−/−^ mice, are subfertile, and sperm from *Acr*^−/−^;*Prss21*^−/−^ mice are unable to fertilize the eggs in vitro [24]. These findings indicate that optimal action of serine proteases on the sperm is required for a normal sperm function in the fertilization process.

In addition to its effect on sperm function, AEBSF could also negatively impact egg quality or the fertilization process itself. In our in vivo fertilization experiment, the highest concentration of AEBSF (i.e., at 5 mM) carried over to the fertilization drop was approximately 0.05 mM. Even though, at 0.1 mM AEBSF, we did not observe a significant sperm cytotoxicity, it is possible that eggs might be more sensitive than the sperm with the presence of AEBSF and this could contribute to reduced fertilization rate.

Based on the findings from in vivo fertilization*,* fertilization rates after AEBSF treatment were higher than the number of pups/plug. This indicates that AEBSF may also impact post-fertilization processes, including preimplantation embryo development, uterine receptivity, and other uterine functions that are crucial for pregnancy. Sun et al. showed that AEBSF significantly reduced embryo–uterine implantation in mice [25]. Therefore, disruption of uterine receptivity could be another contraceptive target of AEBSF in addition to the effect on sperm motility and function. We also found that N9 did not completely prevent in vivo fertilization, suggesting that there were some sperm that were able to migrate to the ampulla to fertilize the eggs or that N9 also had an impact during the post-fertilization period in addition to its spermicide activity. Therefore, the follow-up experiments will address the effect of AEBSF on egg quality during and after fertilization process in vivo.

When assessing the toxicity in vitro in human ECT cells, we found that AEBSF at doses of 1 and 5 mM significantly decreased cell viability. However, cell toxicity after AEBSF (1 and 5 mM) treatment was not as severe as that of N9-treated cells. Moreover, our results from the toxicity evaluation in mouse vagina showed that AEBSF at 25 mM (~5 times higher concentration than the treatment in the sperm motility and IVF assays) was not toxic to mouse vaginal epithelial cells when tested in vivo. These data suggest that (1) epithelial cells lining the lower reproductive tract of mouse and human respond differently to AEBSF and (2) the effect of the difference between in vivo and in vitro microenvironment must be taken into account when performing toxicity test.

N9 was originally marketed as an anti-infective agent, a contraceptive method that functions to protect against STIs [26]. Historical studies suggest that N9 damages the cell membranes of various STI pathogens, such as gonorrhea, chlamydia, and HIV, effectively working as a microbicide in the same manner it functions as a spermicide [26]. Over time, additional evidence showed that N9 displays its destructive effects on the female reproductive tract, causing physical irritation, inflammation, and damage to the integrity of the cervical and vaginal epithelia [27–31]. Through these inflammation and epithelial cell destruction, N9 in turn allows for greater STI transmission, contradicting its original utility [10, 11]. Our study shows that the protective barrier of the vagina is not affected upon vaginal use of AEBSF compared to N9, at least in female mice. N9 displayed a trend of greater cell death upon acute and short-term exposures (both in mice and human ECT cell lines). Therefore, while N9 may function as a microbicide, its secondary effects on the vaginal epithelium deem it unjustifiable for use.

Additionally, N9 is correlated to a disruption in vaginal flora after repeated use, allowing for overgrowth of anaerobic bacteria and suppression of aerobic bacteria, following the logic of its original application as a microbicide [32]. Specifically, N9 limits the number of *lactobacilli* present in the vagina, creating a dysbiosis that can lead to a number of vaginal conditions including bacterial vaginosis and clinical candidiasis [27]. When developing a contraceptive, it is important to consider the impact on the vaginal microflora as this could have consequences on both STIs and natural infection development. For protease inhibitor to be a superior alternative to N9, it must not generate these same effects on the vaginal microbiome. Further studies evaluating STI risk upon exposure to protease inhibitors (such as AEBSF) and their effects as a potential microbicide should be performed in vivo as well as in the human vaginal 3D culture [33] in order to mimic vaginal microenvironment in women.

More pressing, we found that the contraceptive effects of N9 were not temporary. After use of N9 in the fertility study was ceased, female fecundity did not return to normal levels. In contrast, female fecundity returned to “before the treatment levels” after discontinuing AEBSF treatment. This data suggests that our prototype compound, AEBSF, is a reversible contraceptive, ideal to be further developed as OCT option, and that its currently marketed counterpart (N9) may not be ideal as originally thought.

In summary, our findings provided a proof-of-concept that the protease inhibitor, AEBSF, acts as a reversible contraceptive in an in vivo mouse model. AEBSF functions through the inhibition of semen liquefaction and partly acts as a spermicide that inhibits sperm motility and leads to a defective sperm function. AEBSF significantly reduces fecundity rate in mice but is qualitatively less damaging to the cervical and vaginal epithelial cells compared to N9. Protease inhibitors remain a prime target to be explored in the development of an intravaginal, pre-intercourse suppository contraceptive gel for women and are worthy of future investigations.

## Methods and materials

### Ethics statement

All animal handling protocols and procedures were carried out according to the Washington State University (WSU) Animal Care and Use Committee guidelines and complied with WSU-approved animal protocols #6151 and 6147. Studies were performed with mice that were housed in a temperature- and humidity-controlled room with access to water and food ad libitum.

### Animals

Adult wild-type (WT) C57B6/J female mice (the Jackson Laboratory, Bar Harbor, ME) were used in all experiments. As the female mice between 2- and 9-month-old are in their reproductive age, the fecundity test was carried out starting at 2-month-old (8-week-old) and ending approximately at 8 months of age. Female mice were singly housed and allowed to mate overnight with a C57BL6/J proven breeder male. Female mice (2- to 3-month-old) were used for the toxicity studies.

### Chemicals and reagents

4-(2-Aminoethyl)benzenesulfonyl fluoride (AEBSF) was purchased from the EMD Millipore Corp., Billerica, MA. Gynol II (containing 3%, equivalent to 49 mM, Nonoxynol 9 (or N9), Options, Madison, NJ) was used as a positive control. Natrosol 250 HX Pharm Hydroxyethylcellulose (HEC) gel was purchased from Ashland, Inc. (Covington, KY). N9 (1467950) for in vitro experiments was purchased from Sigma-Millipore (St. Louis, MO). 3-(4,5-Dimethylthiazol-2-yl)-2,5-diphenyltetrazolium bromide, M6494) (MTT) was purchased from Thermo Fisher Scientific (Waltham, MA). Dimethyl sulfoxide (DMSO, D128) was purchased from Fisher Scientific (Hampton, NH).

### Transcervical injection of compounds to evaluate the effectiveness of fecundity in female mice in vivo

HEC gel was prepared in 0.85% normal saline and sorbic acid as previously described [34]. HEC gel, a known universal placebo for contraceptive gels, was used as a negative control because the consistency of HEC gel mimics human cervicovaginal fluid at a pH of 4.4 and does not disrupt sperm or vaginal epithelia [34–37]. AEBSF was dissolved in 0.85% normal saline prior to mixing with HEC gel. AEBSF at a dose of 300 μg/mouse was used in this in vivo experiment. This is approximately equivalent to 25 mM based on the information that the molecular weight of AEBSF is 239.7 g/mol and that AEBSF mass in each treatment/application is 300 μg in a total volume of 50 μL HEC gel (or 6 mg/mL).

We previously showed that AEBSF at 300 μg effectively inhibits the sperm transport to the oviduct in mice [19]. Additionally, the acidity of HEC gel and a low pH in the reproductive tract [38] will maintain the inhibitory activity of AEBSF for more than 24 h [39].

There were three phases to this study: “before,” “during,” and “after” where the litter size (number of pups born) and number of pups per plug were recorded throughout the experiment. Prior to any treatment, female mice were mated with fertile male mice to determine a baseline fecundity ensuring that the mice used in this study followed a normal fertility pattern, called “before.” After the validation of female fertility, fertile females were randomly assigned into three groups: (1) HEC gel (vehicle control), (2) AEBSF, and (3) N9. To mimic the biological circumstance, by which the women would use this contraceptive intermittently throughout their reproductive period, the treatment was performed every 4–5 days as the following.

The estrous cycle was evaluated at 8:00 a.m. daily in all groups using a vaginal smearing method as previously indicated [40]. Female mice in estrus were transcervically injected with either HEC gel (50 μL), AEBSF (300 μg in 50 μL HEC gel), or 49 mM N9 (50 μL of Gynol II) using a 24-gauge IV catheter (BD InSyte Autoguard, Becton, Dickinson and Company, Warwick, RI) at 3:00 p.m.

The IV catheter device serves similar to a nonsurgical embryo transfer device [41]. Immediately after the treatment, female mice were individually housed and bred overnight with fertile males. The following morning after mating if a copulatory plug at the vaginal opening was observed, the female was designated as 0.5 days post-coitus (dpc). Regardless of a copulatory plug, female mice were separated from the males to ensure no additional mating without the use of a treatment gel. The number of pups per plug was also determined “during” the treatment. Once the mice had two litters in the “during” phase, they transitioned to the “after” phase where they were mated with male mice without the use of contraceptive/treatment gels. The mice were then allowed to have two litters in this phase. Any pups resulting from the experiment were culled. At the end of the experiment, animals were euthanized using CO_2_ asphyxiation with cervical dislocation, and the reproductive tracts were collected for histological analysis (*n =* 4–7 mice/treatment/time-point).

### Fertilization rate in vivo

To determine the fertilization rate in vivo, adult female mice were superovulated using a protocol previously published [19]. Immediately after human chorionic gonadotropin injection, female mice were treated with HEC, AEBSF, or N9 using transcervical injection as indicated above. Females were then mated with a proven male breeder overnight. The next morning, the presence of copulatory plug was determined, and females were euthanized at 11:00 a.m. Fertilized eggs were collected from the ampulla of the oviduct and determined by the presence of two pronuclei (zygotes). In vivo fertilization was quantified as the (number of zygotes/total ovulated eggs)^*^100 (*n =* 4–7 mice/treatment).

### Assessment of sperm motility and viability

Cauda epididymides were collected from C57B6/J male mice (3- to 6-month-old) in Leibovitz’s L15 media (Invitrogen, Carlsbad, CA) as described previously [42]. Sperm were isolated and incubated with different doses of AEBSF (0, 0.001, 0.01, 0.1, 1, and 5 mM) or 49 mM N9 in human tubal fluid (HTF; Sigma-Millipore), supplemented with 3 mg/mL bovine serum albumin (BSA) in 5% CO_2_ at 37 °C. Following 1-h treatment, the sperm motility was assessed using sperm computer analysis (SCA) as recommended by manufacturer’s protocol (Fertility Technology Resources, New Orleans, LA). At least 10 imaged areas were used for data analysis per treatment (*n =* 4–5 mice/treatment), and at least 500 sperm were analyzed per treatment per mouse. After 2 h of AEBSF treatment, sperm viability was assessed. Briefly, sperm from different treatment groups were incubated with 250 nM of YOYO-1 (Y3601, Invitrogen) for 10 min at room temperature. YOYO-1 was used as cell-impermeant nucleic acid stain, allowing the dye to permeate and stain the dead cells. Sperm were pelleted down by centrifugation (3000 *g*, 5 min), washed twice with phosphate-buffered saline (PBS), and later fixed with 4% paraformaldehyde (PFA) for 20 min at room temperature. PFA was then washed off twice with PBS, and sperm were resuspended in 20 μL of PBS, spread onto slides, and cover-slipped with ProLong Gold with DAPI (Invitrogen). Fluorescent signals of YOYO-1 and DAPI were assessed at 400× magnification using Olympus IX51 fluorescent microscope (Olympus, Center Valley, PA). The percentage ratio of sperm with green fluorescent signal (YOYO-1 stained representing dead sperm) to the total sperm (DAPI-positive) was calculated. At least 10 imaged areas were used for data analysis (*n =* 4–5 mice/treatment, 203–352 sperm analyzed/treatment/mouse).

### Assessment of sperm function

IVF was used to determine the effect of AEBSF on sperm function. Sperm was collected from the cauda epididymis and incubated with various doses of for 1 h as described above. The sperm concentration during preincubation is ~ 1–5 × 10^4^ sperm/μL, depending on each male. Approximately 1 × 10^5^ sperm/mL were used in the fertilization drop with COCs collected from C57B6/J females. Therefore, the highest volume of preincubating sperm containing AEBSF was ~ 1 μL in a final 100-μL fertilization drop. To collect the COCs, adult females were superovulated with gonadotropins as previously described [42]. Six to eight hours after the fertilization, the presence of male and female pronuclei was determined and designated as fertilized eggs or zygotes and, then, two-cell embryos the following morning. The percentages of zygotes were calculated as a ratio of total number of eggs at the beginning of the fertilization (*n =* 31–83 eggs/treatment condition from a total of 3–5 female mice).

### Priming of vaginal epithelial cells using Depo-Provera

To determine the effect of AEBSF on the cervical and vaginal mucosa, adult C57B6/J female mice were subcutaneously injected with 2.5 mg medroxyprogesterone acetate (MDPA; Depo-Provera, Amphastar Pharmaceuticals, Inc., Rancho Cucamonga, CA) in 100 μL sterile normal saline) to mimic the diestrus-like state, at which the stratified squamous layer is minimally present [20]. As a result, the cervical and vaginal epithelia are lined with living epithelial cells, instead of keratinized layers. This is a well-accepted protocol suitable for testing a cervical and vaginal barrier susceptibility to toxicants and pathogens [9, 43, 44].

### Evaluation of cervical and vaginal toxicity in mice in vivo

Seven days after MDPA treatment, 50 μL of (1) HEC gel, (2) AEBSF (300 μg), or (3) N9 was injected directly into the vaginal tract adjacent to the cervix using a 24-gauge IV catheter as described above. Staining of YOYO-1 dye in the vaginal canal was performed using methods previously described [9]. Briefly, treatment gels were left for 10 min, or injections were repeated daily for 3 consecutive days. At the end of the treatment, YOYO-1 dye at a dose of 5 μM in 80 μL PBS was injected into the vagina. After 15 min, the vagina was rinsed with PBS and the animal was euthanized. The reproductive tract was collected and immediately imaged on a fluorescent stereomicroscope (Leica MZ10f, Leica Microsystems, Buffalo Grove, IL). Vaginal tissue samples were cut opened longitudinally to the uterus to reveal the inner epithelial layers. Images of green fluorescent signal (representing vaginal epithelial cell death) were taken and quantified using ImageJ software (NIH Bethesda, MA). Data were normalized to HEC gel. A no-treatment control was also included in this experiment as a baseline evaluation to determine the effect of the procedure on vaginal cell death.

### Evaluation of cell viability in human ectocervical cells in vitro

Human ectocervical cells (Ect1/E6E7 or ECT, CRL-2614) were purchased from the American Type Culture Collection (ATCC; Manassas, VA). ECT cells were cultured in the keratinocyte serum-free media (KSFM; Invitrogen) as recommended by the ATCC. Cells were plated and left to adhere overnight in 96-well plates at a density of 25 000 cells/well in 200 μL of KSFM. Experiments were performed in triplicates and repeated three separate times. Twenty-four hours after plating, media were replaced with 200 μL of KSFM containing 0, 0.001, 0.01, 0.1, 1, and 5 mM of AEBSF or 49 mM N9. MTT assay was used to assess the cell viability. MTT was prepared at 5 mg/mL in PBS and then filtered sterile. Twenty microliters of MTT was added into each well at 1 or 3 h of treatment and incubated for 4 additional hours at 37 °C, 5% CO_2_. Media containing MTT were carefully aspirated and discarded. DMSO (150 μL) was added into each well to solubilize formazan crystals. The absorbance was quantified using Epoch microplate reader (BioTek, Winooski, VT) at 560 nm. The cell viability for each treatment group was calculated by the percentage compared to the control condition (untreated cells), which is defined by (treatment readout/0 mM readout)^*^100. Bright-field images were also taken 3 h after the treatment, immediately before MTT addition.

### Statistical analysis

Data were analyzed using a GraphPad Prism version 6.00. All graphs represent the mean ± SEM. All data were evaluated for statistically significant differences (*P* < 0.05) using a one-way or a two-way analysis of variance (ANOVA) with multiple comparisons test.

## Supplementary Material

Supplemental_movie_ioaa049

Supplemental_Still_Figure_ioaa049

## Notes

**Grant Support**: This study was supported by the Washington State University, by the College of Veterinary Medicine Intramural Research Grant, and partly by the Eunice Kennedy Shriver National Institute of Child Health and Human Development of the National Institutes of Health under Award Number R01HD097087.
