# Supplementary figures and images for: Serine protease inhibitor disrupts sperm motility leading to reduced fertility in female mice†
Source: Biol Reprod. 2020 Apr 18;103(2):400–10. doi: 10.1093/biolre/ioaa049 (PMC7401027; doi:10.1093/biolre/ioaa049)

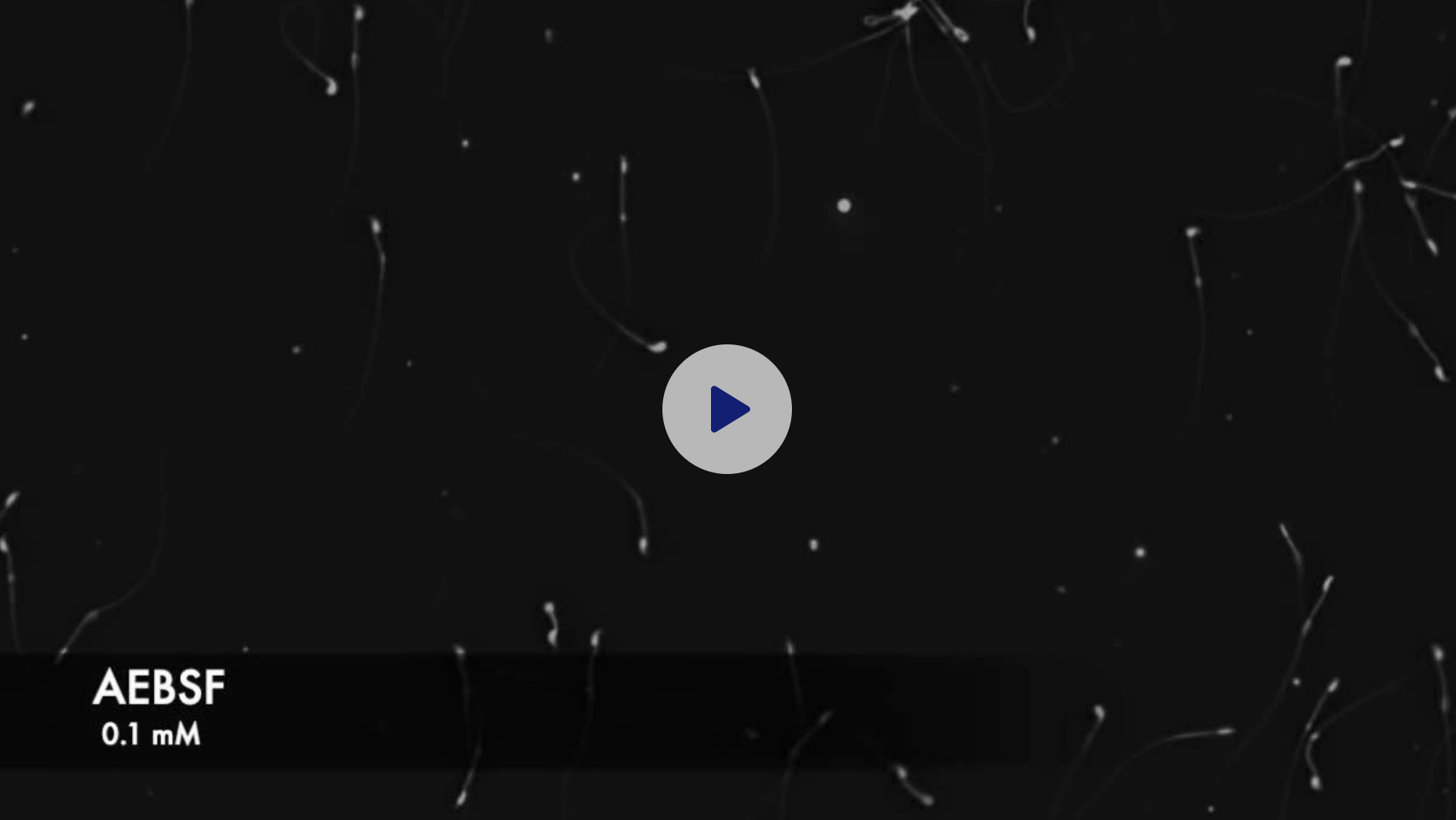

Supplement: Supplemental_Still_Figure_ioaa049 [file supplemental_still_figure_ioaa049.png]
